# Scaling of spike-timing based neuron model for mammalian olfaction with network size

**DOI:** 10.1186/1471-2202-15-S1-P90

**Published:** 2014-07-21

**Authors:** Bolun Chen, Jan R Engelbrecht, Renato Mirollo

**Affiliations:** 1 Physics Department, Boston College, Chestnut Hill, MA 02467, USA; 2 Mathematics Department, Boston College, Chestnut Hill, MA 02467, USA

## 

We investigate extensions to the model put forward by Brody and Hopfield [1] for spike-timing based pattern recognition applied to mammalian olfaction. Their model implements a pattern recognition algorithm realized in the dynamics of a network of coupled IF neurons subject to a sine-wave rhythm. Subsets of these neurons can synchronize through the principle of one-to-one mode locking. Their network represents 3 layers of neural activity, the first two of which are inspired by the connectivity of glomeruli and mitral cells in mammalian olfactory circuits and the gamma-rhythm activity observed in the olfactory bulb. Specifically in this model a pattern of glomerular activity representing a given odor causes a particular subset of model mitral cells to synchronize and this synchronous activity can drive a "grandmother" model cortical cell through threshold triggering a recognition event. In this study we quantify the performance of their original model and compare it to our extensions of the model such as using a network-generated rhythm rather than a sine-wave, introducing inhibitive feedback and generalizing to p-q mode locking strategies. We compute the scaling with respect to the number of mitral neurons of a measure of the number of odor patterns the model can recognize. Quite remarkably we find this performance can increase very fast with increasing network size -- consistent with exponential scaling.
